# Seroepidemiology of *Toxoplasma gondii* amongst Pregnant Women in Jazan Province, Saudi Arabia

**DOI:** 10.1155/2014/913950

**Published:** 2014-11-13

**Authors:** Hussein Aqeely, Eman K. El-Gayar, Darakhshan Perveen Khan, Abdullah Najmi, Ayesha Alvi, Ibrahim Bani, Mohamed Salih Mahfouz, Saif Elden Abdalla, Ibrahim M. Elhassan

**Affiliations:** ^1^College of Medicine, . Jazan University, Saudi Arabia; ^2^Department of Parasitology, College of Medicine, Suez Canal University, Ismailia 41522, Egypt; ^3^College of Applied Medical Sciences, Jazan University, Saudi Arabia; ^4^Department of Obstetrics and Gynecology, Jazan General Hospital, Saudi Arabia; ^5^Center of Biomedical Research, Jazan University, Saudi Arabia; ^6^Department of Microbiology, College of Medicine, Jazan University, Jazan, Saudi Arabia

## Abstract

*Background. Toxoplasma gondii (T. gondii)* is an obligate intracellular protozoan parasite of worldwide distribution. There is limited information about the seroprevalence of toxoplasmosis in the southern area of Saudi Arabia. The current study was carried out to determine the prevalence of *T. gondii* in pregnant women in Jazan province. *Materials and Methods.* The study was conducted between January and June 2013 and included 195 pregnant women, data on sociodemographic and predisposing factors were collected from each participant. Venous blood samples were collected following standard operating procedures. Serological analysis for latent toxoplasmosis (levels of IgG) and active toxoplasmosis (IgM) was done using Enzyme Linked Immunosorbent Assay (ELISA). *Results.* The overall seroprevalence of *T. gondii* in the study area was 24.1%. The seroprevalence of anti-*Toxoplasma* IgG was 20% (39 out of 195), whereas IgM seropositivity was 6.2% (12 out of 195). Only 4 pregnant women tested positive for both IgG and IgM. The highest IgG and IgM seroprevalence was among the study participants aged 35 to 39 years (13.5% and 35.1%, resp.). The seropositivity rate of *T. gondii*-specific antibodies was higher among pregnant women from the urban areas than those from rural communities (7.4% versus 0% and 21% versus 15.4% for IgM and IgG, resp.). *Conclusions.* The seroprevalence of *T. gondii* was high in pregnant woman in Jazan. The prevalence of toxoplasmosis increases with increase of age. Awareness health education program in Jazan needs to be maintained and developed to targeted pregnant women.

## 1. Introduction

*Toxoplasma gondii* is an obligate intracellular opportunistic protozoan parasite that can infect any nucleated cells of different vertebrate hosts including humans [1]. It was estimated that one third of the world's population is infected by* T. gondii* [2]. Studies show that about 90% of infections in immune competent humans are asymptomatic while up to 10% presented with a flue like with cervical lymphadenopathy or ocular disease. Infection during pregnancy may cause a wide range of clinical manifestations in the offspring depending on the gestational age, when maternal infection was acquired, immunologic development of the fetus, and the virulence of the parasite [3]. In immunosuppressed patients, toxoplasmosis can cause severe encephalitis by acute infection or reactivation of latent infection [3]. Acute and latent infections during pregnancy are commonly diagnosed by the detection of anti-*T. gondii*-specific IgG and IgM antibodies and by the avidity of* T. gondii*-specific antibodies [4].

Epidemiological studies suggest that prevalence of* T. gondii* infection in pregnant women varies substantially among different countries; in Europe it varies from 9% to 63%, 63.2% in Germany [5], 19.8% in Italy [6], and 9.1% in the UK [7]. In Asian countries the seroprevalence of toxoplasmosis was reported low: 3.7% in Korea and 11.2% in Vietnam [8, 9] while prevalence is as high as 41.6% to 45% in Indian pregnant women [10], 66.9% in Jordan and 53.1% in Kuwait [11, 12]. In the American continent, the seroprevalence of toxoplasmosis was reported to be 77.5% in Brazil [13] and 63.5% in Colombia [14].

In spite of the fact that toxoplasmosis is one of the diseases distributed worldwide, there is scarce information on the prevalence and epidemiology of the disease in the Arabian Gulf countries. Only few studies have been conducted to determine the prevalence of* T. gondii* in Saudi Arabia, including studies carried out in Riyadh, the Eastern Region, and Jeddah [15–17]. To our knowledge the level of the transmission, epidemiology, and prevalence of toxoplasmosis in Jazan area have not previously been reported. Therefore, we conducted a cross sectional study to determine the prevalence of* T. gondii* infection in pregnant women in Jazan province, Kingdom of Saudi Arabia, and to determine the characteristics of the study population that was associated with the infection.

## 2. Materials and Methods

### 2.1. Study Area and Participants

This study was conducted in Jazan province (also called Jizan, Gizan, or Gazan), which is situated on the coast of the Red Sea. It lies in the southwest corner of Saudi Arabia and directly north of the border with Yemen. It is populated by less than a one million residents. Like most coastal towns, it is highly populated with cats. The majority of these cats live very close to human settlements and restaurants feeding on left-over food in the garbage bins and by food supplied by the locals. Only a small percentage of the cat population is kept as domestic pets by some individuals.

### 2.2. Study Design and Sampling Procedures

This study is an observational cross-sectional descriptive study based on the values *π* = 0.5 (no previous estimate of prevalence of* T. gondii* in Jazan province), desired marginal error = 0.075 and *z* or (confidence level 95%) = 1.96; nonresponse rate 10% of the study sample size was estimated at 200 women. Systematic random sampling procedure was utilized to select women within the antenatal clinic.

### 2.3. Data Collection

Data was collected using a structured questionnaire composed of 45 questions relating to demographic clinical data and other information such as age, general knowledge on toxoplasmosis, obstetrical history, house type, and standard of living. Information on potential risk factors such as the presence of cats or kittens, direct contact with cats or cat boxes, eating behavior, kitchen hygiene, history of blood transfusion, and history of eye disease were also collected from each study subject.

### 2.4. Sample Collection and Laboratory Processing

Five mL of venous blood was collected aseptically from 195 pregnant women during the period between January and June 2013 in Jazan General Hospital. The blood samples were then transported to the laboratory of the Medical Research Center, Jazan University. Then, serum was separated from the whole blood by centrifugation at 3000 rpm for ten minutes at room temperature. The separated serum was labeled and kept at −20°C until use. Finally, it was tested for anti-*T. gondii* IgG and IgM antibodies using ELISA test kit (Human Gesllschaft for biochemical and diagnostic, Max Plank, Germany) following the manufacturer's instruction.

### 2.5. Data Management and Analysis

Data collected by the study team was verified and entered at the Jazan Faculty of Medicine. Data was entered and analyzed using the SPSS software (version 17.0). Descriptive statistics (frequencies, cross tabulation and percentages) were used for summarizing the dependent and outcome variables. Pearson's chi-square/Fisher Exact test was used to assess the differences among proportions. Univariate analyses were conducted to identify factors associated with* Toxoplasma* IgG as a dependent variable and a set of selected explanatory variables. Variables that were associated with* Toxoplasma* IgG were included in the stepwise backward likelihood multivariate logistic regression. Adjusted odds ratios (ORs) and their 95% confidence intervals were reported. Hosmer-Lemeshow statistics were used to evaluate the goodness of the fit of the model. All statistical tests were two-sided, and *P* value <0.05 was used to indicate statistical significance.

## 3. Results

### 3.1. Characteristics of the Study Subjects

The questionnaire data revealed that 81.4% of participants have never heard or seen information about toxoplasmosis prior to the interview. Also, 95.1% participants were unaware of being serologically tested or not for toxoplasmosis.

The blood analyses were successfully carried out for 195 subjects (97.75%) from the target of 200 women. More than 50% of pregnant women were in the age group of 20–29 years. The distribution of educational status showed that 65.6% of women had completed secondary education and above. One hundred sixty two (83.1%) participants were living in urban areas and 74.4% of them were housewives. Fertility history showed that 30.3% of the women had no childbearing experience and 36.4% of them had one child to two children, while women with five or more children were 17.4%.

### 3.2. Prevalence of IgG and IgM Antibody Responses

The overall seroprevalence of* T. gondii* among the pregnant women was 24.1%. Out of the 195 pregnant women examined for seroprevalence of* T. gondii*, 12 (6.2%) (95% C.I. (3.6–10.5)) and 39 (20.0%) (95% C.I. (15.0–26.1)) were positive for* Toxoplasma* IgM and IgG, respectively. Of the 12 women with IgM titers, four also had IgG titers. Only 4 women (out of 12 women) were positive for both IgG and IgM. The highest seroprevalence of* Toxoplasma* IgM was found to be 13.5% among pregnant women in the age group (35–39). The highest prevalence of* Toxoplasma* IgG was reported among the same age group 35.1%. Seroprevalence of* Toxoplasma* IgM was 0%, 5.5%, and 6.4% for first, second, and third trimester, respectively; the seroprevalence of Toxoplasma IgG was 16.7%, 21.8%, and 17.3% for first, second, and third trimester, respectively.

### 3.3. Level of Seroprevalence of* Toxoplasma* IgM and IgG Antibodies according to Some Selected Characteristics

There was no significant difference (*P* = 0.6) in* Toxoplasma* seropositivity among individuals reported to have domestic cats. Women who reported presence of domestic cats were found to be 20% compared to 19.7% among women who did not have domestic cats. There was a highly significant association between seropositivity among participant coming from urban and rural areas (*P* = 0.00).

Over 90% of the study participants reported to have a habit of eating out, of which 21.1% were* T. gondii* seropositive. However, there was no significant association (*P* = 0.1) between habit of eating nonhome cooked meals and* T. gondii* seropositivity. There was no significant difference (*P* = 0.1) in* Toxoplasma* seropositivity among working and nonworking individuals or different occupations (Table 1).

### 3.4. Risk Factors Associated with* Toxoplasma gondii* IgG among Studied Women

The results of the univariate and multivariate logistic regression analyses for potential risk factors of* Toxoplasma* IgG are shown in (Table 2). Univariate analysis revealed that only increase in age (25–34) (OR = 3.75, *P* < 0.05) had a corresponding increase with distribution of the infection. Variables like washing kitchen daily, eating raw or undercooked minced meat, and eating outside of the home showed increased odds ratio (1.96, 2.01, and 3.5) of infection, respectively, but without significant associations (*P* value > 0.05 for all). The multivariate analysis further suggested the same results of univariate analysis.

## 4. Discussion

In this study, we report for the first time the seroprevalence of* T. gondii* in the southern part of Saudi Arabia among immunocompetent pregnant women. Primary infection of* T. gondii* acquired during pregnancy could be passed to the fetus and might lead to serious consequences including abortion or neurological disorder and visual impairment [18]. Previous studies have shown that more than 80% of the congenitally infected infants are asymptomatic at earlier age of life; however, some of them develop manifestations at childhood and adolescence. The seroprevalence of* T. gondii* in Jazan province is 24.1% which is in line with the results of the pervious cross sectional studies carried out in other regions in Saudi Arabia and neighboring Gulf countries, showing that the prevalence of* T. gondii* ranges between 25 and 36% [16, 17, 19–21]. Low prevalence rates of 10% were reported in the United Kingdom [7], Korea [8], and Norway [22] and as high as 77.5% in Brazil [13] and 63.5% in Colombia [14]. Higher prevalence rates were also reported in some neighboring Arab countries like Jordan 66.9% and Kuwait 53.1% [11, 12]. The difference in prevalence rate between different countries can be explained by variation of geographical and climatic conditions between different areas as the success of oocysts sporulation better in hotter and wetter areas [23].

The high seroprevalence of* T. gondii* in the Jazan area is expected with the presence of a relatively high population of cats. This study showed no significant association between* T. gondii* infection and presence of domestic cats and this corroborates with studies reported in Nigeria [24], India [25], and Tanzania [26]. On the other hand, studies from Ethiopia [27], Taiwan [28], and France [29] showed a significant association between contact with cats and prevalence of* T. gondii*. In the present study, presence of stray cats near the household was reported by the majority of the study subjects (80%); oocysts of* T. gondii* is usually not found on cat fur [30] but are found hidden in soil along with cat feces. Since contact with soil is difficult to avoid especially in Jazan which is characterized by dusty winds (*Qabra*) from time to time, this might explain the high prevalence of toxoplasmosis in Jazan area.

As IgM is considered an indicator for recent/active infection, our data revere that active cases are approximately 3 times lower than inactive infections. The highest seroprevalence of* T. gondii* IgM and IgG antibodies was observed in the group of women of old age. This is in accordance with the data obtained from previous studies carried out in different geographical settings [26, 27]. This may be an indicator of high infection risk at early adolescences; also older individuals have higher susceptibility to be exposed to many risk factors for* T. gondii* infection during their lives than younger individuals. On the other hand, Al-Qurashi et al. have carried-out a study in eastern region of Saudi Arabia and they reported that the prevalence of toxoplasmosis declined with age [16].

This study showed a highly significant association between seroprevalence in pregnant women from urban areas compared to rural areas. This can be due to high income of urban areas and their different habits in eating poultry and junk food from restaurants which have been found to be a major source for* T. gondii* transmission [31]. The absence of a statistically significant relationship between the prevalence of* Toxoplasma* infection among pregnant women in Jazan and many of the risk factors explored in the study—such as physical contact with cats, eating raw, or undercooked minced meat-tasted raw meat while preparing food and drinking unfiltered water—does not rule out that these factors have no effect on the transmission of Toxoplasmosis. However, it may suggest that such factors play a limited role in this region due to certain religious, cultural, and eating behavioral characteristics [26, 32].

## 5. Conclusions

A high proportion of* T. gondii* in human infections is generally asymptomatic and self-limiting. This study showed that the majority of pregnant women did not know about the disease and it is deleterious effect on pregnancy and the fetus; there is a need for implementation and maintenance of control activities in the area, including developing of health education programs targeting pregnant women and immunocompromised individuals to reduce the risk of acquiring toxoplasmosis infection. At the same time, there is a strong and obvious need to control urban stray cat population to reduce the risk of transmission of the parasite. Although our data will not provide a complete picture of prevalence of* T. gondii* infections in the entire population of Jazan area, it clearly showed that* T. gondii* is prevalent in the study area. Large scale epidemiological study to determine the prevalence of* T. gondii* in the entire region of the southern part of Saudi Arabia is needed as infections are more common in the abovementioned study area.

## Figures and Tables

**Table 1:** Seroprevalence of *Toxoplasma* IgM and IgG according to selected characteristics (*n* = 195).

Characteristics	Prevalence	95% CI	*P* value
*Toxoplasma* IgM			
Age groups (*n* = 12)			
15–19	0 (0.0)	—	0.633
20–24	3 (7.1)	2.6–19.1	
25–29	3 (5.0)	1.8–13.7	
30–34	1 (2.6)	0.61–13.2	
35–39	5 (13.5)	6.0–28.1	
40–44	0 (0)	—	
Residence type (*n* = 12)			
Urban	12 (7.4)	4.3–12.5	0.000
Rural	0 (0)	—	
Nationality (*n* = 12)			
Saudi	9 (6.0)	3.2–11.0	0.552
Non-Saudi	3 (6.7)	2.4–17.9	
Work status (*n* = 12)			
Working	1 (2.0)	0.48–10.4	0.138
Not-working	11 (7.6)	4.3–13.1	
Trimester of pregnancy (*n* = 10)			
1st trimester	0 (0)	—	0.545
2nd trimester	3 (5.5)	2.0–14.9	
3rd trimester	7 (6.4)	3.2–12.6	
Overall prevalence	**12 (6.2)**	**3.6–10.5**	

*Toxoplasma* IgG			
Age groups (*n* = 39)			
15–19	1 (11.1)	2.5–44.5	0.150
20–24	5 (11.9)	5.3–25.1	
25–29	12 (20.0)	11.9–31.8	
30–34	6 (15.4)	7.3–29.3	
35–39	13 (35.1)	21.4–51.4	
40–44	2 (25.0)	7.4–56.8	
Residence type (*n* = 38)			
Urban	34 (21.0)	15.4–27.9	0.538
Rural	4 (15.4)	6.3–33.7	
Nationality (*n* = 39)			
Saudi	28 (18.7)	13.3–25.7	0.256
Non-Saudi	11 (24.4)	14.3–38.8	
Work status (*n* = 12)			
Working	6 (12.0)	5.7–23.9	0.072
Not-working	33 (22.8)	16.7–30.3	
Trimester of pregnancy (*n* = 34)			
1st trimester	3 (16.7)	6.1–39.6	0.760
2nd trimester	12 (21.8)	13.0–34.4	
3rd trimester	19 (17.3)	11.4–25.4	
Overall prevalence	**39 (20)**	**15.0–26.1**	

**Table 2:** Results of multivariate logistic regression analysis of IgG related factors.

Variable	Univariate analysis			Multivariate analysis		
	OR	95% C.I.	*P* Value	OR	95% C.I.	*P* Value
Pregnancy stage						
1st trimester	1			1		
2nd trimester	1.04	0.27–3.9	0.950	0.69	0.15–3.10	0.629
3rd trimester	0.75	0.33–1.6	0.480	1.02	0.24–4.33	0.977
Age groups						
15–24 years (ref.)	1			1		
25–34	3.75	1.30–10.7	0.014	3.95	1.19–13.08	0.024
35–49	2.25	1.00–5.02	0.048	2.14	0.85–5.36	0.103
Working status						
Yes (ref.)	1					
No	2.16	0.85–5.52	0.107	1.178	0.64–4.91	0.263
Residence						
Rural (ref.)	1					
Urban	1.46	0.47–4.53	0.511	2.88	0.61–13.67	0.182
Washing kitchen daily						
Yes (Ref.)	1					
No	1.96	0.87–4.43	0.104	2.25	0.92–4.47	0.740
Eating raw or undercooked minced meat						
No (Ref.)	1					
Yes	2.01	0.17–22.8	0.572	—	—	—
Eating outside of the home						
No (Ref.)	1					
Yes	3.5	0.44–27.4	0.237	3.63	0.41–32.3	0.249

Hosmer and LemeshowTest, Chi square = 6.253, *P* value = 0.66.
